# *In Vivo* Imaging of [60]Fullerene-Based Molecular Spherical Nucleic Acids by Positron Emission Tomography

**DOI:** 10.1021/acs.molpharmaceut.3c00370

**Published:** 2023-08-02

**Authors:** Antti Äärelä, Tatsiana Auchynnikava, Olli Moisio, Heidi Liljenbäck, Putri Andriana, Imran Iqbal, Jyrki Lehtimäki, Johan Rajander, Harri Salo, Anne Roivainen, Anu J. Airaksinen, Pasi Virta

**Affiliations:** † Department of Chemistry, University of Turku, FI-20500 Turku, Finland; ‡ Research and Development, Orion Pharma, FI-20380 Turku, Finland; § Turku PET Centre, University of Turku, FI-20520 Turku, Finland; ∥ Turku Center for Disease Modeling, University of Turku, FI-20520 Turku Finland; ⊥ Accelerator Laboratory, Åbo Akademi University, FI-20520 Turku, Finland; # Turku PET Centre, Turku University Hospital, FI-20520 Turku, Finland

**Keywords:** molecular spherical nucleic acids, PET-imaging, nanoparticles, delivery

## Abstract

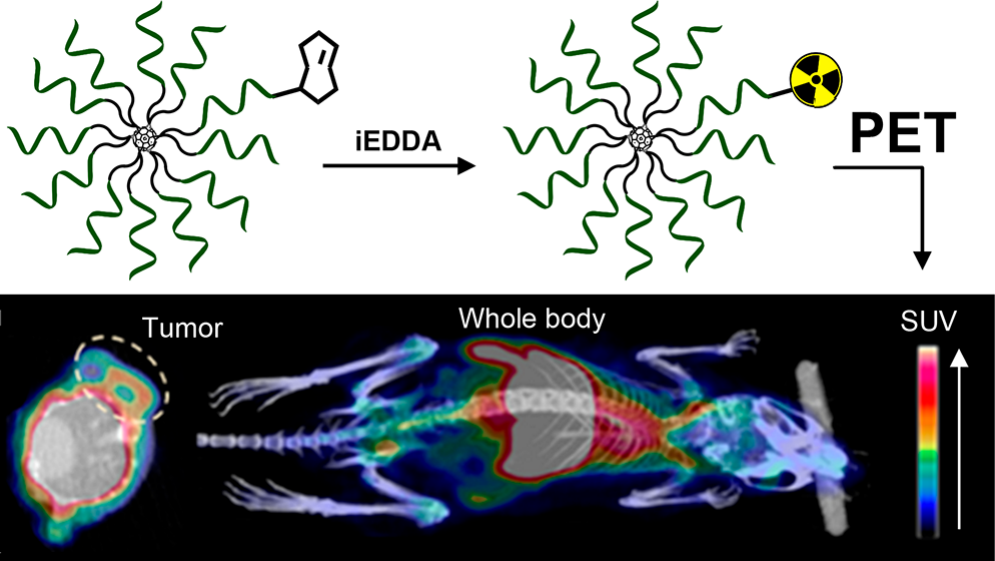

^18^F-Labeled [60]fullerene-based molecular spherical nucleic acids (MSNAs), consisting of a human epidermal growth factor receptor 2 (HER2) mRNA antisense oligonucleotide sequence with a native phosphodiester and phosphorothioate backbone, were synthesized, site-specifically labeled with a positron emitting fluorine-18 and intravenously administrated via tail vein to HER2 expressing HCC1954 tumor-bearing mice. The biodistribution of the MSNAs was monitored *in vivo* by positron emission tomography/computed tomography (PET/CT) imaging. MSNA with a native phosphodiester backbone (MSNA-PO) was prone to rapid nuclease-mediated degradation, whereas the corresponding phosphorothioate analogue (MSNA-PS) with improved enzymatic stability showed an interesting biodistribution profile *in vivo*. One hour after the injection, majority of the radioactivity was observed in spleen and liver but also in blood with an average tumor-to-muscle ratio of 2. The prolonged radioactivity in blood circulation may open possibilities to the targeted delivery of the MSNAs.

## Introduction

Spherical nucleic acids (SNAs) are dendritic nanoparticles, consisting of appropriate core material (gold,^1^ silica,^2^ liposomes,^3^ proteins)^4^ and a dense oligonucleotide (ON) layer. SNAs have beneficial properties, which may be utilized for the delivery of therapeutic ONs.^5−7^ They show efficient free cellular uptake via class A scavenger receptor-mediated endocytosis (correlates with the density and chemistry of the component ONs),^8−10^ resistance to nuclease-mediated degradation,^11,12^ and prevented renal clearance (depending on particle size and ability to form a protein corona).^13,14^ The radial formulation may also be beneficial together with the covalent conjugation strategy:^15^ The role of the cell/tissue-specific ligands may be emphasized on the outer sphere of the SNAs, which at the same time hide unfavorable biodistribution properties of the loaded ON content.^16^ The studies addressing *in vivo* biodistribution of SNAs are limited to a few promising examples considering polydisperse lipid- and gold core-based SNAs.^1,13,14,17−20^ They are able to penetrate critical biological barriers, such as the blood–brain barrier and blood–tumor barrier^21−23^ leading to clinical progress that has been made to deliver SNAs to glioblastoma for therapeutic gene regulation.^24^ DNA containing nanoparticles show usually strong accumulation in liver and spleen after intravenous (i.v.) administration.^14,21,25,26^ In general, i.v.-administered nanoparticles accumulate in organs rich in cells of the mononuclear phagocyte system (MPS), such as liver, spleen, and lungs.^27−29^

In contrast to high valency polydisperse SNAs based on gold and lipid nanoparticles, low-valency molecular spherical nucleic acids (MSNAs), assembled on a [60]fullerene core, may not activate scavenger receptors as strongly, as the recognition correlates with the ON density.^2,30^ This reduces cellular uptake but might also prevent accumulation in organs responsible for the phagocyte system (e.g., Kupffer cells in liver), which may be beneficial for the systemic delivery. Additionally, MSNAs can be specifically heterofunctionalized with tissue-specific ligands or labeling groups making them interesting tools for therapeutic and diagnostic applications.^31,32^

**Scheme 1:**
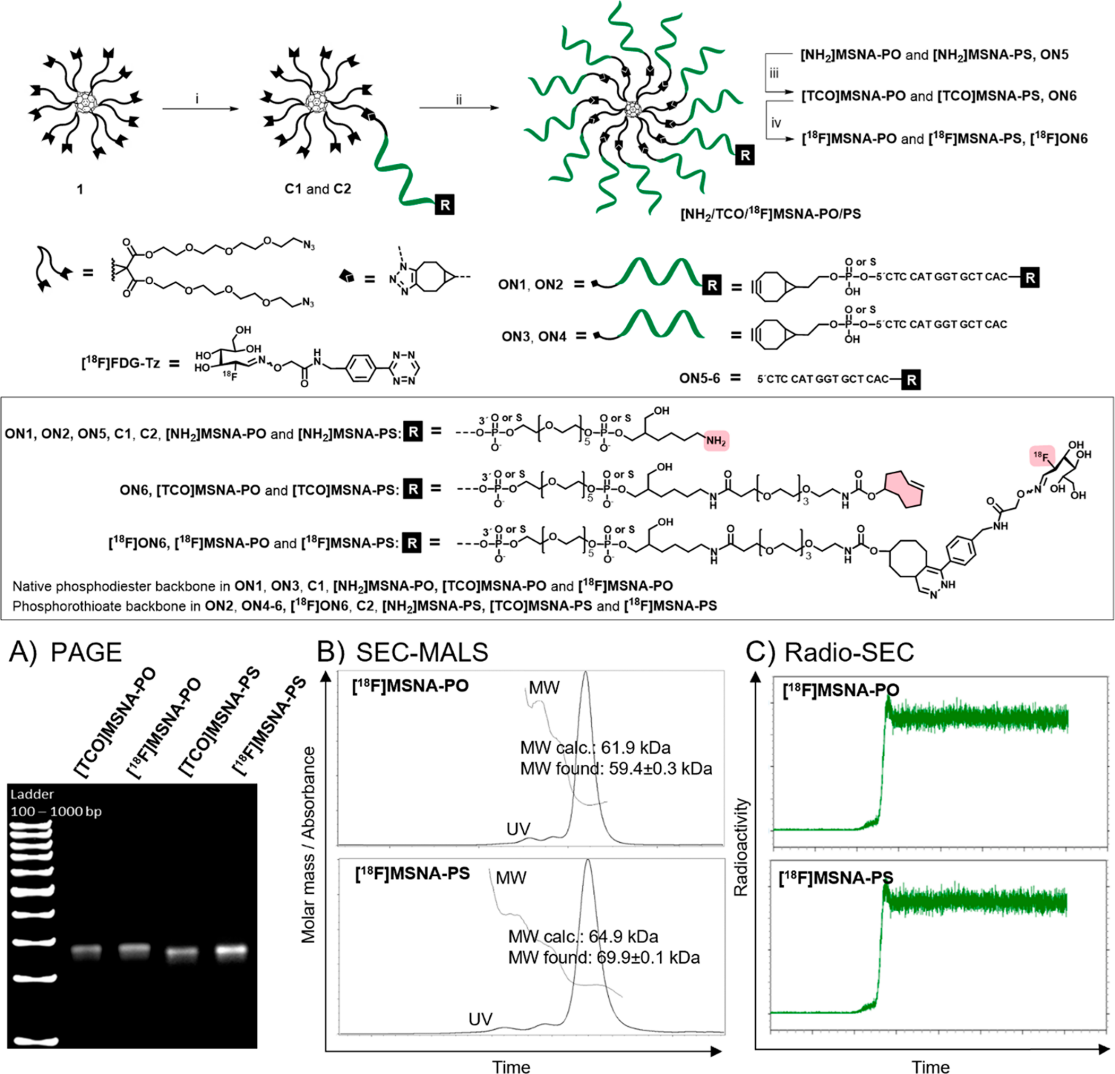
Synthesis of Radiolabeled MSNAs Conditions: (i) BCN-modified oligonucleotide **ON1** or **ON2**, C_60_ core **1** (4 equiv) in DMSO, overnight at r.t., (ii) **C1** or **C2**, BCN-modified **ON3** or **ON4** (1.2 equiv/arm) in aqueous 1.5 M NaCl, 3 days at r.t., (iii) TCO-PEG_4_-NHS ester, 0.1 M sodium borate (pH 8.5), 4h at r.t., (iv) **[**^**18**^**F]FDG-Tz** in PBS (pH 7.4), 5 min, r.t. Characterization of MSNAs: (A) Polyacrylamide gel electrophoresis, (B) size-exclusion chromatography (SEC) equipped with a multiple angle light scattering (MALS) detector was used for molecular weight estimation of **[**^**18**^**F]MSNA-PO** and **[**^**18**^**F]MSNA-PS**, (C) Radio-SEC **[**^**18**^**F]MSNA-PO** and **[**^**18**^**F]MSNA-PS**.

**Figure 1:**
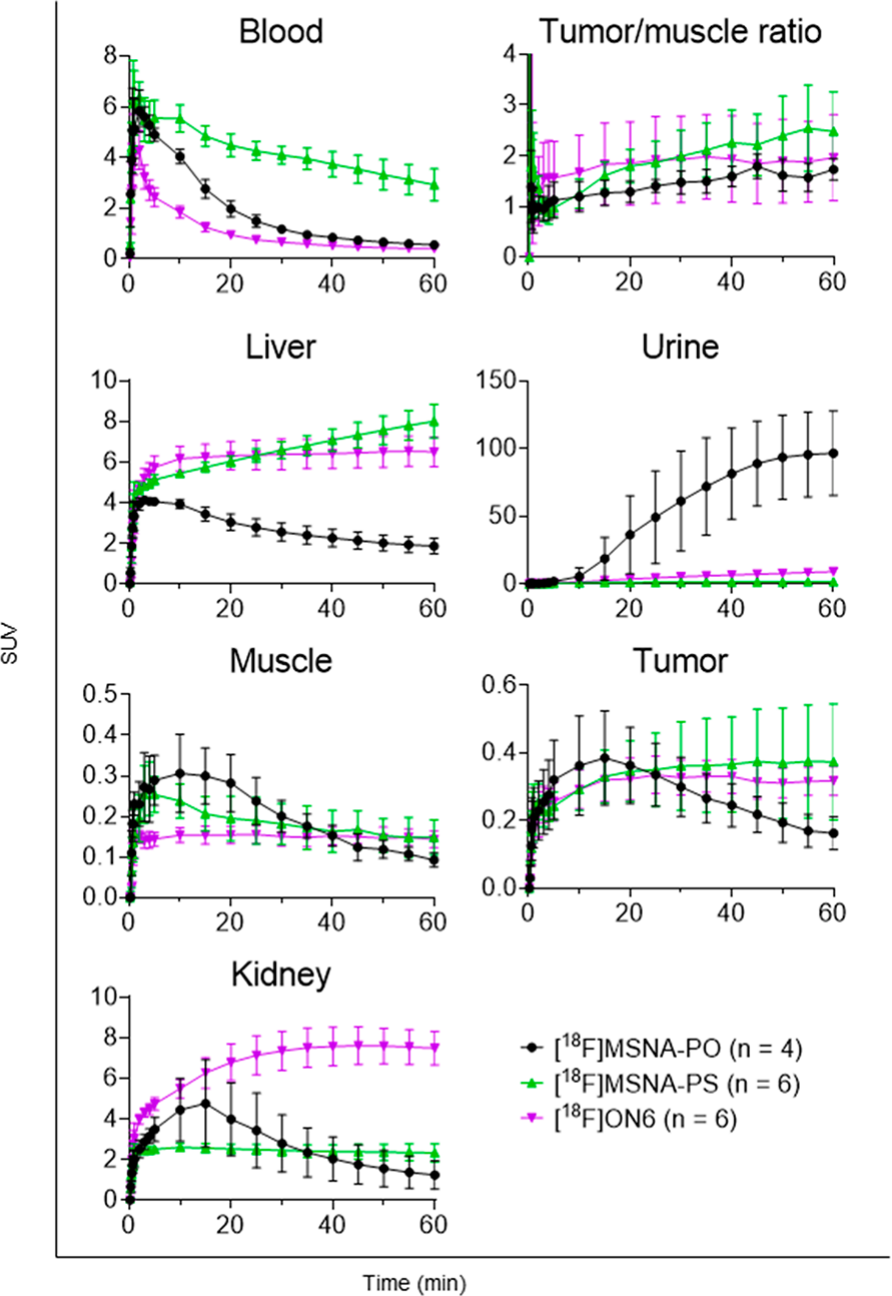
*In vivo* distribution kinetics of [^**18**^**F]MSNA-PO**, **[**^**18**^**F]MSNA-PS**, and **[**^**18**^**F]ON6** in HCC1954 tumor-bearing female mice shown as time-activity curves for blood, tumor/muscle ratio, liver, urine, muscle, tumor, and kidney expressed as standardized uptake value (SUV).

**Figure 2:**
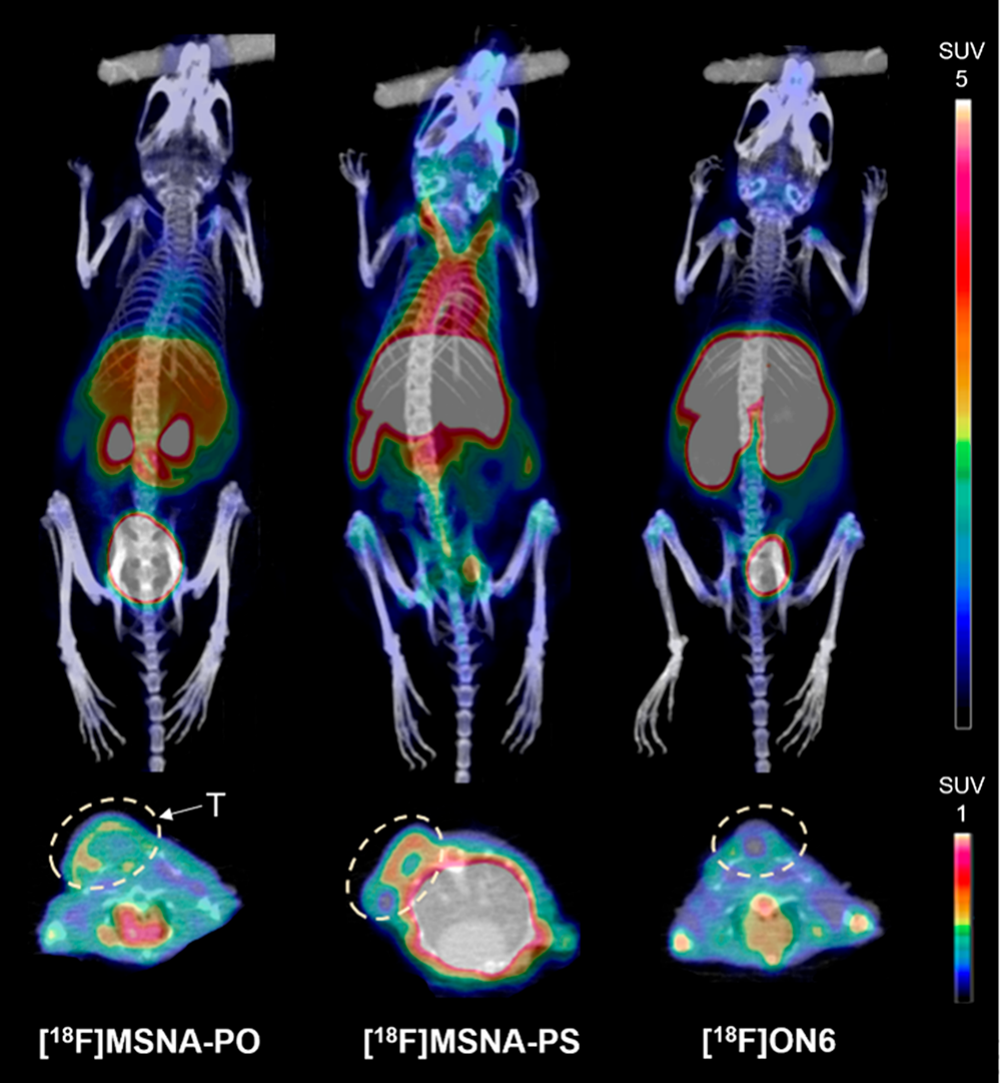
Maximum intensity projection coronal (top) and axial plane (bottom) PET/CT images at 15–60 min postinjection of **[**^**18**^**F]MSNA-PO**, **[**^**18**^**F]MSNA-PS**, and **[**^**18**^**F]ON6** in HCC1954 tumor-bearing female mice. T denotes tumor.

In this work, we demonstrate site-specific introduction of radiolabel to [60]fullerene-based MSNAs and provide preliminary information on how low-valency MSNAs behave *in vivo.* The described controlled attachment of only one radiolabeling group minimizes the label’s effect on the biodistribution properties of the MSNA. To study the effect of the backbone chemistry on the biodistribution, two 12-armed [60]fullerene-based MSNAs consisting of a human epidermal growth factor receptor 2 (HER2) mRNA antisense oligonucleotide sequence with both native phosphodiester (**MSNA-PO**) and phosphorothioate (**MSNA-PS**) backbone were synthesized, specifically monolabeled with fluorine-18 (Scheme 1), i.v.-administrated via tail vein into HCC1954 tumor-bearing mice and imaged by positron emission tomography/computed tomography (PET/CT) (Figures 1 and 2). The ON sequence selected for this study has been previously used as a model sequence in the assembly of SNAs.^2^ It has been shown to downregulate HER2, overexpression of which is associated with various cancer types as it promotes malignant cell growth and differentiation.^33,34^ HER2 downregulation has been shown to inhibit proliferation and induce apoptosis in HER2 expressing cells.^35−38^ The antisense activities of **MSNA-PS** and **MSNA-PO** were confirmed *in vitro* by Western blot analysis using human breast cancer (BT-474) cells. For the site-specific monoradiolabeling, our recently published two-step procedure for the assembly of [60]fullerene-based MSNAs^31^ and inverse electron-demand Diels–Alder (iEDDA) cycloaddition with a 2-[^18^F]fluoro-2-deoxy-d-glucose-labeled tetrazine, **[^18^F]FDG-Tz**,^39,40^ were utilized. The homogeneity and authenticity of the radiolabeled MSNAs were verified by different analytical methods, and they were successfully traced *in vivo* with PET/CT imaging. Single-stranded PS ON was traced as a comparison to study the effect of the MSNA formulation to the biodistribution of ONs.

## Materials and Methods

### General Remarks

Reagents were purchased from Sigma-Aldrich (St. Louis, MO), except 6-methyl-tetrazine-5-FAM ((4-(6-methyl-1,2,4,5-tetrazin-3-yl)phenyl)methanamine-5-fluorescein) and *trans*-cyclooctene (TCO)-PEG_4_-NHS ester (2,5-dioxopyrrolidin-1-yl) 3-[2-[2-[2-[2-[[(4*Z*)-cyclooct-4-en-1-yl]oxycarbonylamino]ethoxy]ethoxy]ethoxy]ethoxy]propanoate were purchased from Jena Bioscience (Jena, Germany) and used as received. No-carrier-added ^18^F-fluoride was produced in-house with a CC18/9 medical cyclotron (Efremov Institute of Electrophysical Apparatuses, St. Petersburg, Russia) from Hyox-18 ^18^O-enriched water (≥97%) purchased from Rotem Industries Limited (Arava, Israel). The compounds were analyzed by nuclear magnetic resonance spectroscopy (NMR, 400 MHz Bruker Avance NMR-spectrometer), ESI-TOF mass spectrometry (Bruker micrOTOF, Bremen, Germany), radio-thin-layer-chromatography (radio-TLC, TLC Silica gel 60 F_254_, Merck, Darmstadt, Germany), and radio high-performance liquid chromatography (radio-HPLC) utilizing UV-detector and radiodetector (Hitachi LaChrom Elite, Schaumburg; radiodetector Bicron Corp, Torrington, CT) and Jupiter Proteo reversed-phase C18 column (4 μm, 250 mm × 4.6 mm; Phenomenex, Torrance, CA) and Protein-Pak 33SW column (7.5 × 300 mm; Waters, Milford, CT). After run, TLC plates were opposed to phosphor imaging plates (BAS-TR2025, Fuji Photo Film Co. Ltd., Tokyo, Japan) for autoradiography detection, scanned with BAS-5000 scanner (Fujifilm, Tokyo, Japan), and analyzed with AIDA Image Analyzer v.4.19 (Raytest Isotopenmessgeräte, Straubenhardt, Germany). HCC1954 and BT-474 ductal breast cancer cells were purchased from the American Type Culture Collection (ATCC, Manassas, VA). Media and supplements for cell culture were mainly obtained from Gibco (Waltham, MA). Female Rj:Athymic-*FOXn1nu/nu* mice were form Janvier Laboratories (Le Genest-Saint-Isle, France). Matrigel was purchased from Corning (Corning, NY). The organ radioactivities were quantified by measuring with a Triathler 3 in. gamma counter (Hidex, Turku, Finland). PET/CT scans were acquired with Inveon Multimodality PET/CT (Siemens Medical Solutions, Knoxville, TN) and analyzed with Carimas software (version 2.10, Turku PET Centre, Turku, Finland).

### Synthesis of DNA Sequences against HER2 mRNA Transcripts

Oligonucleotides **ON1–ON5** were synthesized by using an automated DNA/RNA synthesizer. A standard phosphoramidite coupling cycle and commercially available 2′-deoxyribonucleotide building blocks were used for the assembly. 3-Phenyl 1,2,4-dithiazoline-5-one (POS) was used as a sulfurization reagent for the synthesis of **ON3–ON5**. The authenticity of the oligonucleotides was verified by MS (ESI-TOF) (electrospray ionization time-of-flight; Table S1 and Figure S1).

### Synthesis of TCO-Modified **ON6**

TCO-PEG_4_-NHS ester (1.5 μmol in 5 μL of dimethyl sulfoxide, DMSO) was added to a buffered mixture of **ON5** (50 nmol in 100 μL of 0.1 M sodium borate, pH 8.5). The reaction mixture was gently shaken for 4 h at room temperature (r.t.) and subjected to RP-HPLC. An analytical RP column (250 × 4.6 mm, 5 μm), a linear gradient from 5 to 35% acetonitrile in 50 mmol L^–1^ triethylammonium acetate over 25 min, a flow rate of 1.0 mL min^–1^, and detection at 260 nm were used for purification. The product fractions were collected and lyophilized to dryness. The authenticity of the product was verified by MS (ESI-TOF) (Figure S2). Isolated yield (55%) of product **ON6** was determined by UV absorbance at 260 nm.

### Synthesis of C_60_-ON Conjugates **C1** and **C2**

Bicyclononyne (BCN)-modified oligonucleotide (**ON1** or **ON2**, 0.2 μmol in 100 μL of H_2_O) was added to a mixture of C60 core **1** (0.8 μmol in 900 μL of DMSO) in a microcentrifuge tube. The reaction mixture was gently shaken overnight at r.t. and subjected to RP-HPLC. A semipreparative RP-HPLC column (250 × 10 mm, 5 μm), a gradient elution from 40 to 100% acetonitrile in 50 mmol L^–1^ triethylammonium acetate over 30 min, and detection at 260 nm were applied. The product fractions were collected and lyophilized to dryness. The authenticity of the products was verified by MS (ESI-TOF) (Figure S3 for **C1** and Figure S4 for **C2**). Isolated yields (40–50%) of **C1** and **C2** were determined by UV absorbance at 260 nm.

### Assembly of **[NH**_**2**_**]MSNA-PO** and **[NH**_**2**_**]MSNA-PS**

C_60_–ON conjugate (**C1** or **C2**, 100 nmol in 200 μL of H_2_O) was mixed with BCN–ON (**ON3** or **ON4**, 1200 nmol in 400 μL of H_2_O), and 257 μL of 5 M NaCl was added. The reaction mixture was gently shaken for 3 days at rt and subjected to RP-HPLC. An analytical RP-HPLC column Aeris 3.6 μm Widepore XB-C18 200 Å, 150 × 4.6 mm (Phenomenex, Torrance, CA), a linear gradient from 5% to 35% acetonitrile in 50 mmol L^–1^ triethylammonium acetate over 25 min, a flow rate of 1.0 mL min^–1^, and detection at 260 nm were used for the purification. The product fractions were collected and lyophilized to dryness. Isolated yields (30–45%) of the products were determined by UV absorbance at 260 nm. The homogeneities of **[NH**_**2**_**]MSNA-PO** and **[NH**_**2**_**]MSNA-PS** were confirmed by polyacrylamide gel electrophoresis (PAGE) (Figure S5).

### Synthesis of **[TCO]MSNA-PO** and **[TCO]MSNA-PS**

TCO-PEG_4_-NHS ester (2 μmol in 10 μL of DMSO) was added to a buffered mixture of the MSNA (40 nmol of **[NH**_**2**_**]MSNA-PO** or **[NH**_**2**_**]MSNA-PS** in 100 μL of 0.1 M sodium borate, pH 8.5), the mixture was gently shaken for 4 h at rt; phosphate-buffered saline (PBS, 450 μL) was added, and the excess TCO-PEG_4_-NHS was removed by centrifugal filtration for 9 min at 14,000 *g* (Amicon Ultra, 30-kDa molecular weight cutoff; Merck, Darmstadt, Germany). The PBS addition and centrifugation were repeated five times. **[TCO]MSNA-PO** and **[TCO]MSNA-PS** were recovered in 90–95% yields after final centrifugation (based on UV absorbance at λ = 260 nm). The authenticity and homogeneity of **[TCO]MSNA-PO** and **[TCO]MSNA-PS** were verified by size-exclusion chromatography equipped with a multiple angle light scattering detector (SEC-MALS) (Figures S6 and S7) and by PAGE (Scheme 1 and Figure S5).

### Polyacrylamide Gel Electrophoresis (PAGE) Analysis of the MSNAs

Native 6% tris-borate-EDTA (TBE) and acrylamide gels were used to analyze the MSNAs’ purity. A precast gel cover (10 × 10 cm in size, Thermo Fisher Scientific, Waltham, MA) was fixed into a vertical electrophoresis chamber, and the chamber was filled with running buffer (90 mM Tris, 90 mM borate, and 2 mM EDTA, 8.3 pH). MSNA samples (5 μL of 0.1 μM MSNA stock mixed with 5 μL of TBE sample buffer) and a DNA ladder (100, 200, and 1000 bp; note, the ladder was only used to confirm the quality and comparability of the runs and cannot be used for size evaluation of the MSNAs) were loaded and electrophoresed at constant 200 V for approximately 30 min. After completion of electrophoresis, gel was removed from the chamber, stained by SYBR Gold Nucleic Acid Stain, and monitored with Gel Doc imaging system (Bio–Rad, Hercules, CA).

### Size-Exclusion Chromatography, Equipped with a Multiple Angle Light Scattering Detector (SEC-MALS), Experiments

SEC-MALS was performed using a 1260 Infinity II HPLC system (sampler, pump, and UV–vis detector; Agilent Technologies, Santa Barbara, CA) equipped with a miniDAWN light scattering detector and Optilab refractive index detector (Wyatt Technologies, Santa Barbara, CA). An AdvanceBio SEC 300 Å 2.7 μm, 4.6 × 300 mm column (Agilent, Santa Clara, CA) and 150 mM sodium phosphate, pH 7.0, as the mobile phase eluting at a rate of 0.2 mL min^–1^ and run time of 20 min were used for each experiment. For each run, 10 μL of sample with a MSNA concentration of 1 mg mL^–1^ in Milli-Q water was loaded onto the pre-equilibrated column. The refractive index was used for the molecular weight calculations using an average refractive index increment (d*n*/d*c*) of 0.1703 mL/g.

### Radiosynthesis of **[**^**18**^**F]FDG-Tz**

**[**^**18**^**F]FDG-Tz** was synthesized with a synthesis sequence starting from commercially available tetra-*O*-acetyl mannose triflate resulting in **[**^**18**^**F]FDG** (2-[^18^F]fluoro-2-deoxy-*d*-glucose), which was purified with semipreparative HPLC to ensure absence of glucose. The glucose-free **[**^**18**^**F]FDG** was conjugated with *N*-(4-(1,2,4,5-tetrazin-3-yl)benzyl)-2-(aminooxy)acetamide via oxime formation and purified with a second semipreparative HPLC. The final product was analyzed by radio-HPLC and radio-TLC. Identity of the final product was confirmed by coinjection with a reference compound. **[**^**18**^**F]FDG-Tz** was synthesized with a radiochemical yield (RCY) of 3.6% ± 0.8 (*n* = 6), molar activity A_m_ of 148.9 ± 37.2 GBq/μmol (*n* = 4) and radiochemical purity (RCP) > 95%. Following conditions were applied for radio-HPLC: Jupiter Proteo C18 column, eluent A: 0.1% trifluoroacetic acid (TFA, *v*/*v*) in water, eluent B: 0.1% TFA (*v*/*v*) in acetonitrile, gradient 15–35% for 0–15 min, 1 mL min^–1^ flow rate. The following conditions were applied for radio-TLC: 95% acetonitrile in 5% water eluent on silica gel TLC plates.

### Determination of the TCO-Loading of **MSNAs**

6-Methyl-tetrazine-5-carboxyfluorescein (8 nmol in 0.8 μL DMSO) was mixed with **[TCO]MSNA-PO** or **[TCO]MSNA-PS** (2 nmol in 8 μL PBS) and the mixture was gently shaken for 2 h at r.t. PBS (450 μL) was added, and the excess 6-methyl-tetrazine-5-carboxyfluorescein was removed by centrifugal filtration for 9 min at 14,000 *g* (Amicon Ultra, 30 kDa). The PBS addition and centrifugation were repeated five times. After the final centrifugation, absorbances at 260 and 492 nm were measured by Nanodrop UV–vis spectrometer (DeNovix, Wilmington, DE) and extinction coefficients of ε = 1,707,942 M^–1^ cm^–^1 for 260 nm and 83,000 M^–1^ cm^–1^ for 492 nm were used to determine the degree of labeling.

### Radiolabeling of **[TCO]MSNA-PO**, **[TCO]MSNA-PS**, and **ON6**

**[**^**18**^**F]FDG-Tz** (50.9 ± 14.7 μL in PBS (pH 7.4), *n* = 5) and **[TCO]MSNA-PO**, **[TCO]MSNA-PS**, or **ON6** (7.6 ± 1.4 nmol in 32.2 ± 7.6 μL, *n* = 5) were mixed. The reaction mixture was kept for 5 min at r.t. The reaction mixture was loaded to Amicon Ultra filter devices (0.5 mL, 30 kDa; Merck, Darmstadt, Germany for **[**^**18**^**F]MSNA-PO** and **[**^**18**^**F]MSNA-PS**, 0.5 mL, 3 kDa, Merck, Darmstadt, Germany for **[**^**18**^**F]ON6**) and filled with additional RNase-free PBS to reach 200 μL. The filters were centrifuged at 14,100 *g* for 5 min at r.t. This procedure was repeated 3 times. The final product was formulated in RNase-free PBS and analyzed with radio-SEC (Waters Protein-Pak, 0.1 M monopotassium phosphate, pH 7.0, 1 mL/min) and radio-TLC. **[**^**18**^**F]MSNA-PO**, **[**^**18**^**F]MSNA-PS**, and **[**^**18**^**F]ON6** were synthesized in 89.6% ± 14.9 RCY (*n* = 8) with RCP > 99%. The authenticities and homogeneities of **[**^**18**^**F]MSNA-PO** and **[**^**18**^**F]MSNA-PS** were verified by SEC-MALS (Figures S8 and S9) and by PAGE (Figures 1 and S5).

### Western Blot

BT-474 cells were grown in Gibco DMEM, low-glucose, GlutaMAX, pyruvate medium (Thermo Fischer Scientific) supplemented with 10% fetal bovine serum (FBS), and 0.07% insulin. The cells were detached from a T75 flask using 0.25% trypsin. After aliquoting the cells to new flasks in 1:3 ratio cell culture was maintained at 37 °C with 5% CO_2_. MSNA bearing the 5′-CTC TCT-3′ sequence was used as a negative control in the Western blotting experiment. Human BT-474 cells were seeded on 6-well plates (150,000 cells/well) 24 h prior to treatment. Medium was replaced with Opti-MEM reduced serum medium (Thermo Fischer Scientific) immediately before treatment with 10 nM **[TCO]MSNA-PO**, **[TCO]MSNA-PS**, or control MSNA. After 7 h of incubation, the medium was replaced with full growth medium, and cells were cultured for another 48 h. Cells were washed twice with PBS and lysed with M-PER Mammalian Protein Extraction Reagent (Thermo Fischer Scientific) on ice. Proteins (10 μg/well) were separated on 4–15% precast polyacrylamide gels and transferred onto nitrocellulose membranes, which were blocked for 2 h in tris-buffered saline (TBS) blocking buffer (LI-COR). Membranes were incubated overnight with mouse monoclonal anti-human HER2 antibody (catalog number #2248, Cell Signaling Technology, Danvers, MA) and mouse monoclonal anti-human β-actin (catalog number #3700, Cell Signaling Technology) diluted in TBS containing 0.1% Tween-20 (1:1,000), followed by three 5 min washes in tris-buffered saline containing 0.05% Tween-20 (TBS-T). Secondary antibody IR-Dye 680RD goat anti-mouse IgG (LI-COR, Lincoln, NE) diluted in TBS containing 0.1% Tween-20 (1:10,000) was incubated with the membrane for 1 h after which excess of secondary antibody was removed with three 5 min washes in TBS-T. Membranes were imaged and protein bands quantified (Figure S10) with LI-COR Odyssey CLx imaging system (LI-COR).

### Animal Experiments

All animal studies were approved by the national Project Authorization Board in Finland (license number: ESAVI/21485/2020) and carried out in compliance with European Union Directive 2010/EU/63 on the protection of animals used for scientific purposes. HCC1954 ductal breast carcinoma cells were cultured in ATCC-formulated RPMI-1640 medium supplemented with 10% FBS and 0.5% penicillin-streptomycin at +37 °C in the presence of 5% CO_2_. Female Rj:Athymic-*FOXn1nu/nu* mice aged 6–8 weeks at the time of cell inoculation were housed in individually ventilated cages under standardized specific pathogen free conditions at the Central Animal Laboratory, University of Turku with a 12 h light/dark cycle and access to standard soy-free food and tap water *ad libitum*. Five million cells in 50% nonsupplemented RPMI-1640 and 50% Matrigel suspension were inoculated subcutaneously in the upper back area of the mice under light isoflurane anesthesia (induction 4–5%, maintenance 1.5–2.5%). Tumor growth was visually monitored for 3–6 weeks, and tumor size (width × length) was measured with an external caliper along with body weight once a week.

### Biological Evaluation and Image Analysis

**[**^**18**^**F]MSNA-PO**, **[**^**18**^**F]MSNA-PS**, **[**^**18**^**F]ON6**, and **[**^**18**^**F]FDG-Tz** in RNase-free PBS were administrated intravenously to tumor-bearing mice (5.3 ± 0.7 MBq in 35–100 μL, *n* = 20). Dynamic 60 min PET imaging was acquired with an Inveon Multimodality PET/CT. PET data obtained in a list-mode were reconstructed with an ordered subsets expectation maximization three-dimensional (OSEM-3D) algorithm into 6 × 10 s, 4 × 60 s, and 11 × 300 s time frames. Quantitative analysis was performed by manually defining regions of interest (ROIs) in tumor, muscle (skeletal), blood pool (heart left ventricle cavity), kidneys, liver, and urinary bladder using CT as an anatomical reference. Time-activity curves were extracted from the 60 min PET data and expressed as standardized uptake value (SUV) versus time after injection. After imaging, animals were sacrificed, organs of interest were harvested, weighed, and measured with γ-counter, and data are presented as percentage of injected radioactivity dose per gram of tissue (%ID/g). Autoradiography and hematoxylin-eosin staining of 20 μm tumor cryosections were performed (Figure S11).

### Statistical Analysis

The statistical analysis and graphical representation of data was carried out using GraphPad Prism (version 9.1.1). Results of the biological evaluation is presented as mean ± standard deviation (s.d.) values. To evaluate statistical significance, an unpaired Student’s *t* test was used, and *p < 0.05, **p < 0.01, ***p < 0.001 probabilities were considered statistically significant.

## Results and Discussion

### Synthesis and Characterization of TCO-Modified MSNAs on the C_60_-Azide Core

Due to the synthetic availability and radially symmetric dense functionalization, hexa adducts of [60]fullerene^41^ have attracted a marked interest as multipodal scaffolds in material and biomedical sciences.^42,43^ Li et al. used azide-modified [60]fullerene core **1** for the assembly of MSNAs for the first time.^2^ They also evaluated the density of ONs and suggested that these 12-armed MSNAs are nearly the smallest possible structures that could lead to Scavenger A receptor-mediated endocytosis. More recently, we optimized the preparation of the [60]fullerene-based MSNAs on **1** and introduced a 2-step SPAAC-based method for the assembly.^31^ In this method, ON-**1** conjugates (cf., **C1** and **C2**) were first synthesized in high yields using BCN-modified ONs and a moderate excess of **1** in organic media (DMSO). The SPAAC-based assembly is then continued with an excess of BCN-modified ONs in aqueous media to yield MSNAs bearing 12 ON sequences. The monofunctionalization can be utilized to introduce labels or ligands specifically to MSNAs. In the present study, the same method was applied. BCN-modified 15-mer DNA sequences against HER2 mRNA transcripts (**ON1**–**ON4**) were synthesized by an automated synthesizer using commercially available phosphoramidite building blocks. This sequence has previously shown antisense activity in SNA formulation against HER2 mRNA transcripts leading to downregulation of HER2,^2^ and we confirmed the antisense activity by Western blot analysis using human breast cancer (BT-474) cells (Figure S10). Our previously reported 2-step process^31^ was used for the assembly of the MSNAs: First, amino-modified **ON1** (PO) and **ON2** (PS) were conjugated via strain-promoted alkyne–azide cycloaddition (SPAAC) with an azide-modified [60]fullerene core (**1**, 4 equiv) in dimethyl sulfoxide (DMSO), which gave C_60_-ON-conjugates **C1** and **C2** in 45–50% isolated yield. Then, **C1** and **C2** were exposed to excess (1.1 equiv/azide arm) **ON3** (PO) and **ON4** (PS) in aqueous 1.5 M NaCl solution. Incubation for 3 days at r.t. resulted in (mono)amino-modified MSNAs. Isolated yields for both **[NH**_**2**_**]MSNA-PO** and **[NH**_**2**_**]MSNA-PS** were 30–45%. To make the MSNAs suitable precursors for radiolabeling, the amino arms of **[NH**_**2**_**]MSNA-PO** and **[NH**_**2**_**]MSNA-PS** were selectively modified via an amide coupling with an excess (100 equiv) of TCO-PEG_4_-NHS in aqueous borate buffer (pH 8.5). The obtained products **[TCO]MSNA-PO** and **[TCO]MSNA-PS** were isolated by centrifugal filtration (30 kDa molecular weight cutoff) with 95% recovery. Homogeneity of the MSNAs was confirmed by PAGE (Scheme 1 A and Figure S5). SEC-MALS was applied to assess the molecular weight of **[TCO]MSNA-PO** and **[TCO]MSNA-PS**: 58.7 ± 0.2 kDa (expected 61.5 kDa) and 66.8 ± 0.5 kDa (expected 64.5 kDa), respectively (Figures S6 and S7). Molecular weights measured by SEC-MALS corresponded relatively well with the calculated values of the 12-arm MSNA species. TCO is prone to isomerization resulting in *cis*-cyclooctene (CCO), which has significantly slower iEDDA reaction kinetics.^44^ Prior to radiolabeling, the amount of reactive TCO was quantified by exposing a sample from **[TCO]MSNA-PO** and **[TCO]MSNA-PS** to a model iEDDA reaction with a tetrazine-modified fluorescent label (6-methyl-tetrazine-carboxyfluorescein). The ratio in absorbances of the obtained fluorescently labeled MSNAs at 260 and 492 nm was used to quantify the yield of the labeling, which was ca. 25% in both cases. This model reaction gave us an estimation of the active TCO content considered in the radiolabeling experiments.

### Synthesis and Characterization of TCO-Modified Single-Stranded Phosphorothioate Oligonucleotide

Phosphorothioate oligonucleotide sequence against HER2 mRNA transcripts **ON5** was synthesized using an automated ON-synthesis on an amino modified solid support. **ON5** was treated with TCO-PEG_4_-NHS in an aqueous borate buffer (pH 8.5) for 4 h and purified by RP-HPLC to yield **ON6** (47%). Authenticity of **ON6** was verified by ESI-TOF (ESI-TOF) (Figure S2B).

### Synthesis and Characterization of ^18^F-Labeled MSNAs and Single-Stranded **ON6**

**[**^**18**^**F]FDG-Tz** was synthesized from tetra-acetylated mannose triflate according to a previously published two-step synthesis sequence with some modifications (Scheme S1).^39,40^**[**^**18**^**F]FDG-Tz** was obtained with a RCY of 3.6% ± 0.8 (*n* = 6), a high *A*_m_ of 148.9 ± 37.2 GBq/μmol (*n* = 4), and a RCP of >95%. The iEDDA reaction between a tetrazine and TCO is known for its bioorthogonality and fast reaction kinetics.^45^ The reaction has been successfully applied for radiolabeling of several different types of nanomaterial-based drug delivery systems in mild reaction conditions,^46−48^ and it is especially well-suited for materials which are sensitive for harsh reaction conditions. Furthermore, the fast reaction kinetics allows its use for labeling with radionuclides with a short physical half-life, such as for fluorine-18 (*t*_1/2_ = 109.7 min). **[TCO]MSNA-PO** and **[TCO]MSNA-PS** and single-stranded **ON6** were efficiently radiolabeled with **[**^**18**^**F]FDG-Tz** (ratio 1:1) in PBS (pH 7.4) by incubating the reaction mixtures for 5 min at r.t. After the reaction, the radiolabeled structures were isolated by centrifugal filtration, yielding **[**^**18**^**F]MSNA-PO**, **[**^**18**^**F]MSNA-PS**, and **[**^**18**^**F]ON6** in high 89.6% ± 14.9 RCY (RCY, *n* = 8) and excellent radiochemical purity (RCP > 99% by radio-HPLC and radio-TLC). According to SEC-MALS the molecular weights of **[**^**18**^**F]MSNA-PO** and **[**^**18**^**F]MSNA-PS** (Figures S8 and S9) were 59.4 ± 0.3 kDa (expected 61.9 kDa) and 69.6 ± 0.1 kDa (expected 64.9 kDa), respectively. PAGE analysis of both [TCO]MSNAs and [^18^F]MSNAs (Scheme 1A) produced single sharp bands, which in conjunction with similar SEC-MALS profiles indicate that the selected radiolabeling methodology did not alter the structural integrity of MSNAs.

### Biological Evaluation

*In vivo* dynamic PET/CT imaging and *ex vivo* biodistribution studies were performed in HCC1954 tumor-bearing female mice to study the MSNAs’ biodistribution and tumor accumulation *in vivo*. **[**^**18**^**F]MSNA-PO**, **[**^**18**^**F]MSNA-PS**, **[**^**18**^**F]ON6**, and **[**^**18**^**F]FDG-Tz** were intravenously administrated via mice tail vein, and the mice were scanned for 60 min with a dedicated small animal PET/CT.

**Figure 3:**
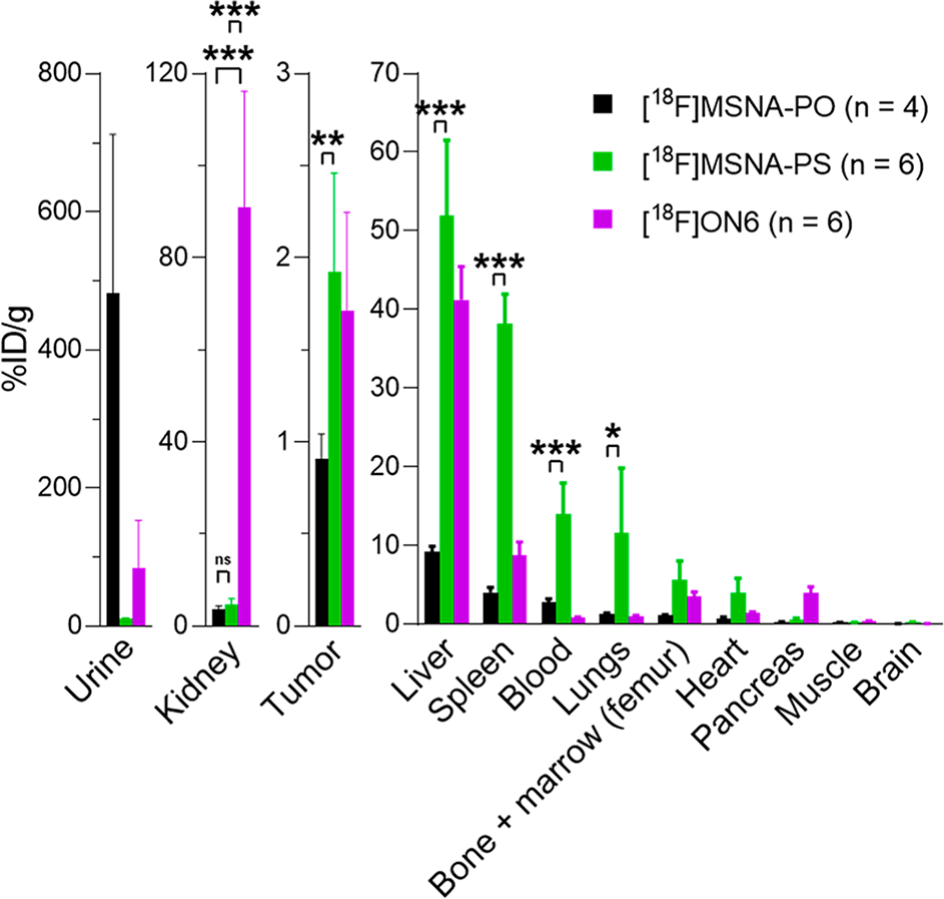
*Ex vivo* biodistribution of **[**^**18**^**F]MSNA-PO**, **[**^**18**^**F]MSNA-PS**, **[**^**18**^**F]ON6** and **[**^**18**^**F]FDG-Tz** in HCC1954 tumor-bearing female mice at 60 min after injection expressed as percentage of injected radioactivity dose per gram of tissue (%ID/g). *p < 0.05, **p < 0.01, ***p < 0.001.

*In vivo* distribution of **[**^**18**^**F]MSNA-PS** (Figure 1) showed significantly prolonged blood circulation compared to that of **[**^**18**^**F]MSNA-PO** with a native PO backbone and to the linear **[**^**18**^**F]ON6**. Standardized uptake value (SUV) of **[**^**18**^**F]MSNA-PS** in blood at 60 min postinjection was 2.93 ± 0.63, which is over 6 times higher compared to the rapidly eliminated profiles of **[**^**18**^**F]MSNA-PO**, and **[**^**18**^**F]ON6**. The obtained data of **[**^**18**^**F]MSNA-PO** are a result of rapid degradation of the PO backbone *in vivo*. This was expected as the protection toward nucleases would need higher density of ONs on the SNAs.^2,49^ The observed improved blood circulation time of **[**^**18**^**F]MSNA-PS** is in the line with previous reports of increased enzymatic stability,^49,50^ increased plasma protein binding,^51−53^ and reduced urinary excretion as a result of PS modification compared to PO backbone (Figure 3). The prolonged circulation of **[**^**18**^**F]MSNA-PS** resulted in a higher accumulation in several organs, including the HCC1954 tumor. The tumor accumulation of **[**^**18**^**F]MSNA-PS** reached maximum at around 45 min with SUV 0.37 ± 0.16 at highest (Figure 1), but the relatively high level of **[**^**18**^**F]MSNA-PS** in blood at 45 min indicates that for better target-to-background ratio even longer imaging time could be advantageous.

Noteworthy, liver accumulation of **[**^**18**^**F]MSNA-PS** remained at a level that is typical for linear PS ONs^50,51,54,55^ (Figures 1 and 3) and clearly less than reported for most nanoparticles.^19−22^ Moreover, increased uptake in the lungs, spleen, liver, bone marrow, and ovaries is well documented for the PS modification (Table S3).^50,56^ Biodistribution of **[**^**18**^**F]FDG-Tz** revealed only minor radioactivity in bone at 60 min after the injection (Figure S12), confirming that the increased bone uptake results from the PS structures itself and not from *in vivo* defluorination. Furthermore, **[**^**18**^**F]MSNA-PS** resulted in a prolonged blood circulation time and significantly lower kidney accumulation compared to the single-stranded **[**^**18**^**F]ON6**. The linear **[**^**18**^**F]ON6** rapidly accumulated and was retained in kidneys with almost 20 times higher uptake (*p* = 0.0004) than **[**^**18**^**F]MSNA-PS**. Despite the quick elimination, **[**^**18**^**F]ON6** exhibited similar but slightly lower tumor level compared to **[**^**18**^**F]MSNA-PS**, making the last one equally good at targeting HER2 mRNA transcripts with beneficially lower kidney uptake. However, slow elimination of **MSNA-PS** could be advantageous for sustained ON delivery and needs to be investigated in future therapeutic experiments.

## Conclusions

Two *trans*-cyclooctene-modified molecular spherical nucleic acids (MSNAs) against HER2 mRNA transcripts were synthesized on a [60]fullerene core with phosphodiester (MSNA-PO) and phosphorothioate (MSNA-PS) backbones. Synthesized MSNAs were site-specifically ^18^F-radiolabeled utilizing biocompatible iEDDA reaction. PAGE and SEC-MALS were used to evaluate the homogeneity and authenticity of MSNAs before and after the radiolabeling. *In vivo* PET/CT was used to study the biodistribution properties of the MSNAs in HER2 expressing HCC1954 tumor-bearing female mice. The biological evaluation revealed the beneficial effect of the PS backbone on the MSNA structure. Liver accumulation of MSNA-PS remained at a level that is typical for linear PS ONs^50,51,54,55^ and clearly less than reported for most other nanoparticular vehicles. Furthermore, a significantly lower accumulation in the kidney, when compared to linear PS ON, was observed. These together resulted in prolonged blood circulation and enhanced accumulation of MSNA-PS to the HER2 expressing tumor tissue. This study serves as a technical demonstration of the applicability of MSNAs to site-specific radiolabeling and *in vivo* tracing providing also a promising benchmark for targeted i.v.-delivery of [60]fullerene-based DNA-nanomaterials, integrated with tissue-targeting small molecular ligands,^57−59^ aptamers,^60,61^ and antibodies.^62^

## Supporting Information Available

ESI-TOF MS characterization of **ON1**–**ON6**, **C1,** and **C2**; PAGE and SEC-MALS of MSNAs; synthesis of radiolabeling agent **[**^**18**^**F]FDG-Tz**; *ex vivo* biodistribution and statistical analysis; autoradiography and hematoxylin-eosin (H&E) staining of 20 μm tumor slices; Western blot analysis of HER2 knock-down (PDF)
