# Supplementary material for: In Vivo Imaging of [60]Fullerene-Based Molecular Spherical Nucleic Acids by Positron Emission Tomography
Source: Mol Pharm. 2023 Aug 2;20(10):5043–51. doi: 10.1021/acs.molpharmaceut.3c00370 (PMC10548468; doi:10.1021/acs.molpharmaceut.3c00370)
Supplement: Supplementary file 1 — mp3c00370_si_001.pdf [file mp3c00370_si_001.pdf]

Supporting information to:

In vivo imaging of [60]fullerene-based molecular spherical nucleic acids by positron emission tomography

Antti Äärelä[a,b], Tatsiana Auchynnikava[a,c], Olli Moisio[c], Heidi Liljenbäck[c,d], Putri Andriana[c], Imran Iqbal[c], Jyrki Lehtimäki[b], Johan Rajander[e], Harri Salo[b], Anne Roivainen[c,d,f], Anu J. Airaksinen[a,c] and Pasi Virta[a]

[a]*Department of Chemistry, University of Turku, FI-20500 Turku, Finland*
[b]*Research and Development, Orion Pharma, FI-20380 Turku, Finland.*
[c]*Turku PET Centre, University of Turku, FI-20520 Turku, Finland*
[d]*Turku Center for Disease Modeling, University of Turku, FI-20520 Turku, Finland*
[e f]*Turku PET Centre, Åbo Akademi University, FI-20520 Turku, Finland*
[f]*Turku PET Centre, Turku University Hospital, FI-20520 Turku, Finland*

Email: pamavi@utu.fi

Table of contents

Table S1 ....S2
Figure S1 ....S3
Figure S2 ....S3
Figure S3 ....S4
Figure S4 ....S4
Figure S5 ....S5
Figure S6 ....S7
Figure S7 ....S6
Figure S8 ....S6
Figure S9 ....S6
Scheme S1 ....S7
Figure S10 ....S8
Table S2 ....S9
Figure S11....................S10
Figure S12....................S11

Structure		MS calc.	MS found
**ON1**	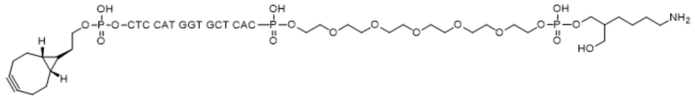	5282.64	5283.06
**ON2**	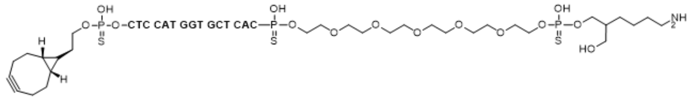	5556.69	5556.24
**ON3**	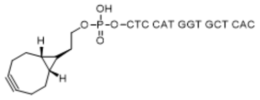	4730.18	4729.86
**ON4**	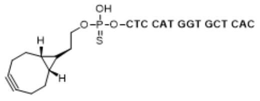	4971.09	4970.70

Table S1. Structures and MS (ESI-TOF) characterization of BCN-modified oligonucleotides used for MSNA assembly. Bold is phosphorothioate oligonucleotide.

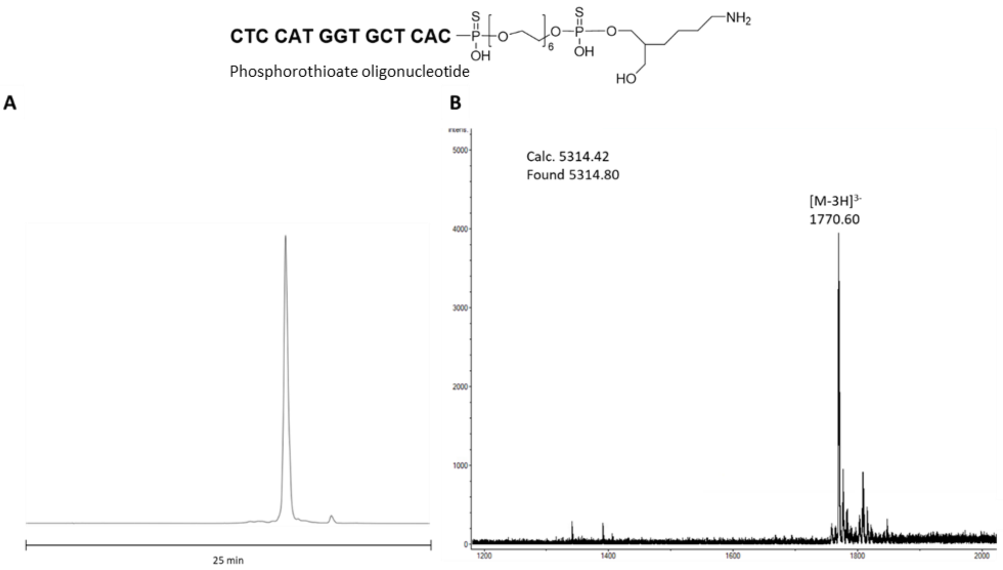

Figure S1. Structure and characterization of phosphorothioate oligonucleotide **ON5**. A) RP-HPLC profile and B) MS (ESI-TOF) spectrum. RP-HPLC conditions: An analytical RP column (250 × 4.6 mm, 5 µm), detection at λ = 260 nm, gradient elution (0–25 min) from 5% to 45% acetonitrile in 50 mM triethylammonium acetate, flow rate 1.0 mL min^{-1}.

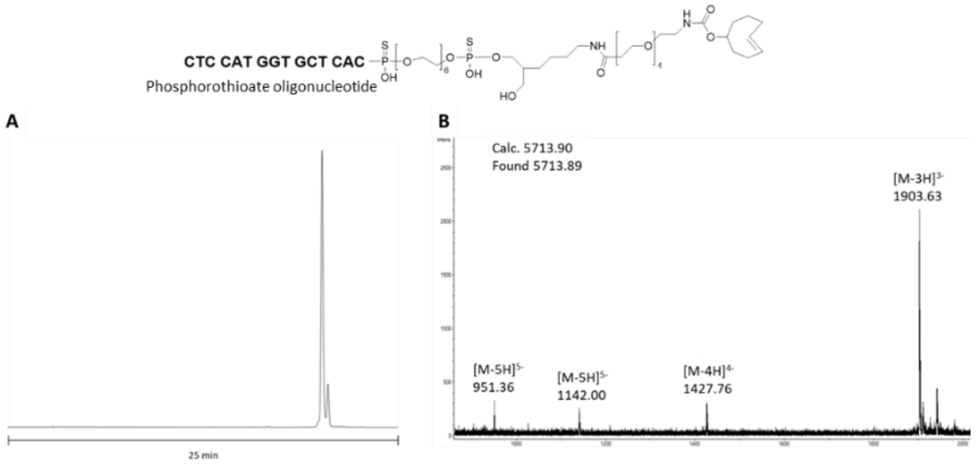

Figure S2. Structure and characterization of phosphorothioate oligonucleotide **ON6**. A) RP-HPLC profile and B) MS (ESI-TOF) spectrum. RP-HPLC conditions: An analytical RP column (250 × 4.6 mm, 5 µm), detection at λ = 260 nm, gradient elution (0–25 min) from 5% to 45% acetonitrile in 50 mM triethylammonium acetate, flow rate 1.0 mL min^{-1}.

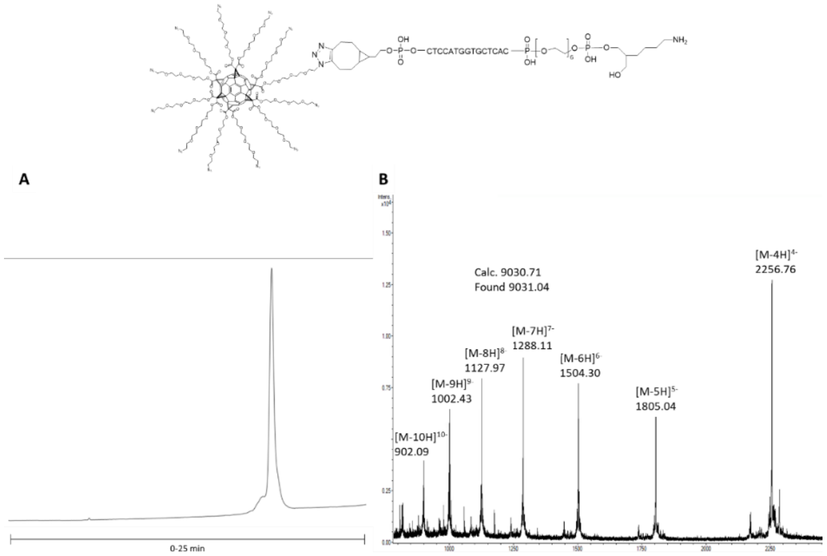

Figure S3. Structure and characterization of C60-ON conjugate **C1** A) RP-HPLC profile and B) MS (ESI-TOF) spectrum. RP-HPLC conditions: An analytical RP column (250 × 4.6 mm, 5 µm), detection at λ = 260 nm, gradient elution (0–25 min) from 40% to 100% acetonitrile in 50 mM triethylammonium acetate, flow rate 1.0 mL min-1.

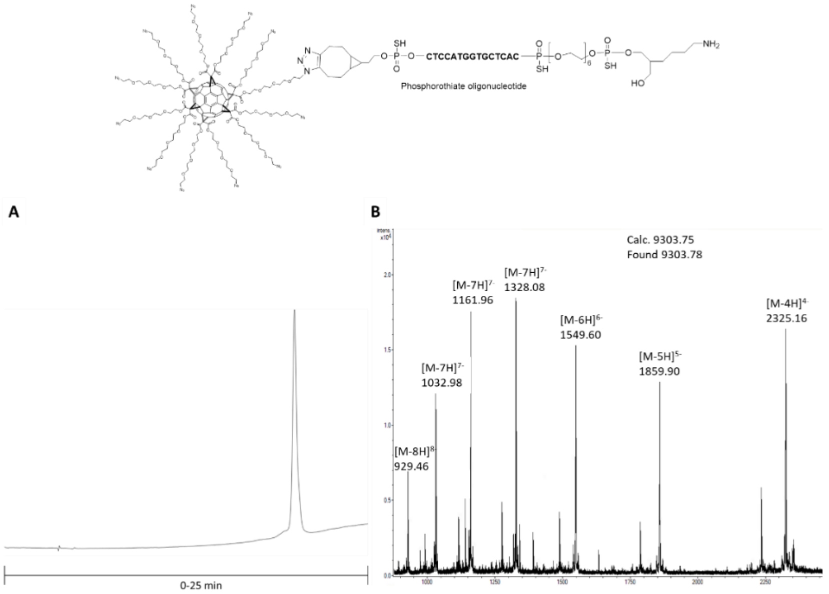

Figure S4. Structure and characterization of C60-ON conjugate **C2** A) RP-HPLC profile and B) MS (ESI-TOF) spectrum. RP-HPLC conditions: An analytical RP column (250 × 4.6 mm, 5 µm), detection at λ = 260 nm, gradient elution (0–25 min) from 40% to 100% acetonitrile in 50 mM triethylammonium acetate, flow rate 1.0 mL min-1.

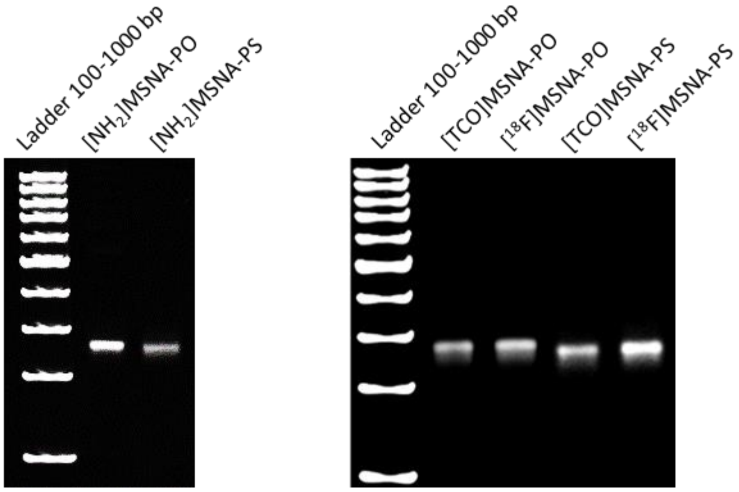

Figure S5. PAGE analysis of MSNAs.

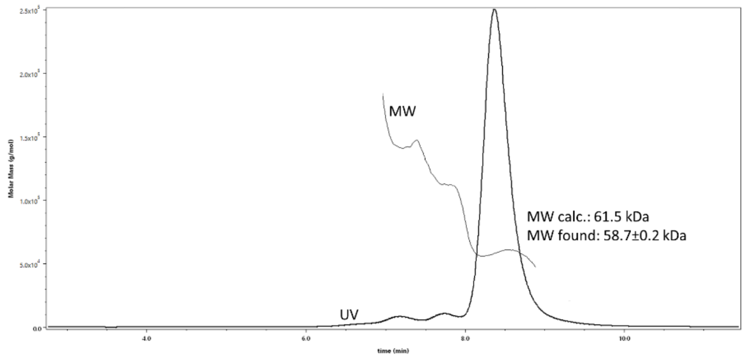

Figure S6. SEC-MALS profile of **[TCO]MSNA-PO.**

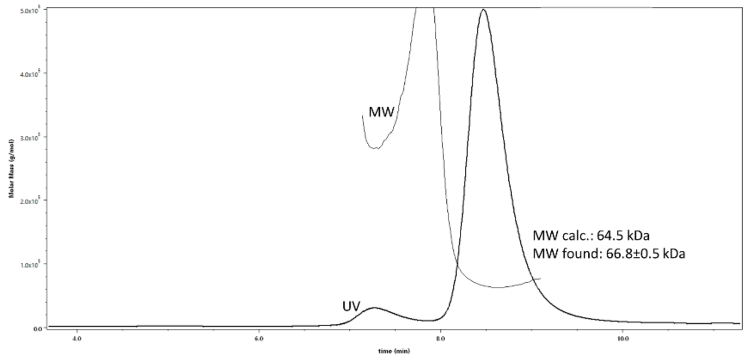

Figure S7. SEC-MALS profile of **[TCO]MSNA-PS.**

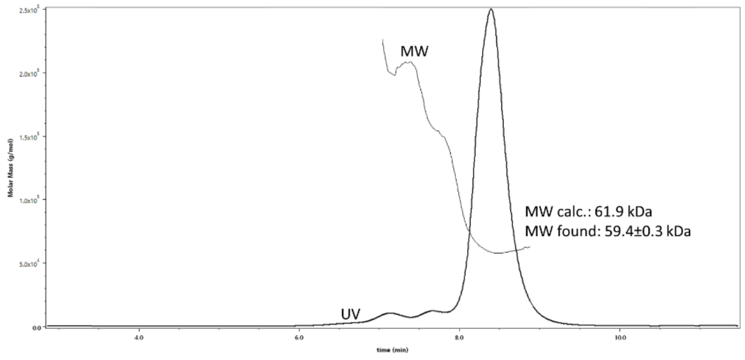

Figure S8. SEC-MALS profile of **[^{18}F]MSNA-PO.**

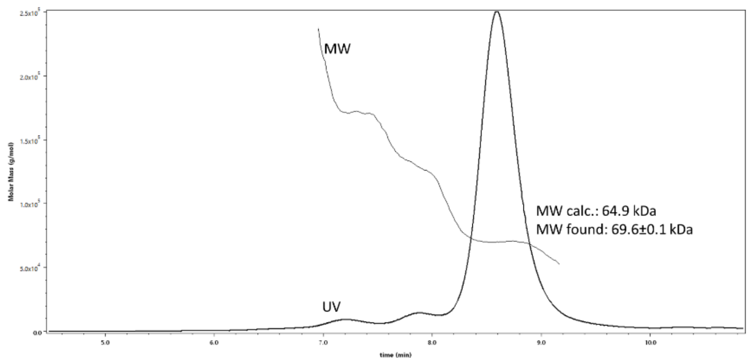

Figure S9. SEC-MALS profile of **[^{18}F]MSNA-PS.**

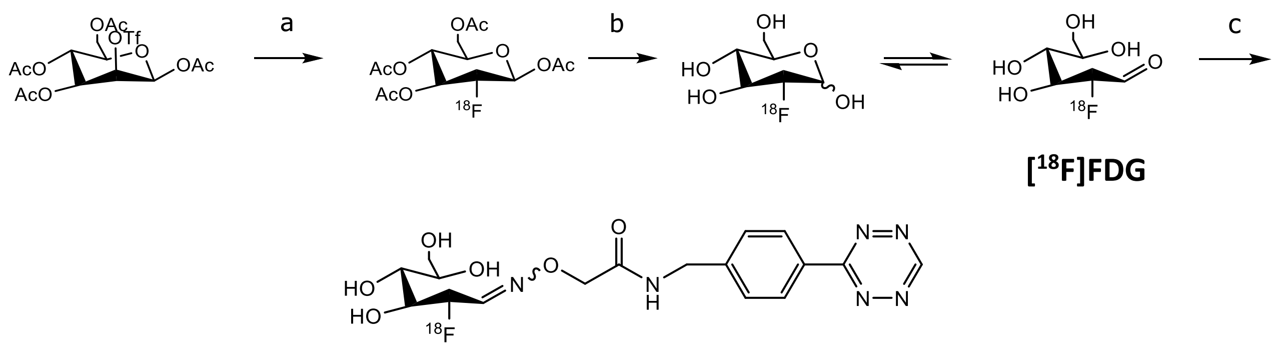

[^{18}F]FDG

[^{18}F]FDG-Tz

Scheme S1. Synthesis of radiolabeling agent **[^{18}F]FDG-Tz**. Reagents and conditions: (a) ^{18}F-K222, K_2CO_3, CH_3CN, 85°C, 5 min, (b) 2 M NaOH, (c) N-(4-(1,2,4,5-tetrazin-3-yl)benzyl)-2-(aminooxy)acetamide, aniliniumacetate-buffer pH 4.6, CH_3CN, MeOH, 75°C, 30 min.

For further details of the synthesis, see:

Keinänen, O.; Partelová, D.; Alanen, O.; Antopolsky, M.; Sarparanta, M.; Airaksinen, A. J. Efficient Cartridge Purification for Producing High Molar Activity 18F-Glycoconjugates via Oxime Formation. *Nucl. Med. Biol.* **2018**, *67*, 27–35. https://doi.org/10.1016/j.nucmedbio.2018.10.001.

Auchynnikava, T.; Äärelä, A.; Moisio, O.; Liljenbäck, H.; Andriana, P.; Iqbal, I.; Li, X.-G.; Virta, P.; Roivainen, A.; Airaksinen, A. Radiolabeling and Biological Evaluation of Functionalized Spherical Nucleic Acids. *Nucl. Med. Biol.* **2022**, pp S39–S40. https://doi.org/10.1016/s0969-8051(22)00117-2.

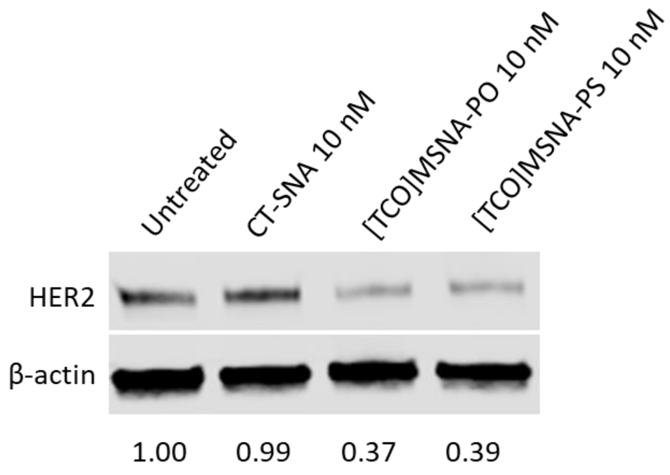

Figure S10. Western blot analysis of HER2 knock-down on BT-474 cells.

Table S2. *Ex vivo* biodistribution and statistical analysis in HCC1954 tumor-bearing female mice 60 min after administration of **[18F]MSNA-PO**, **[18F]MSNA-PS**, **[18F]ON6** and **[18F]FDG-Tz.**

Tissue	%ID/g				Statistical significance (p-value)		
	[18F]MSNA-PO	[18F]MSNA-PS	[18F]ON6	[18F]FDG-Tz	[18F]MSNA-PO vs [18F]MSNA-PS	[18F]MSNA-PO vs [18F]ON6	[18F]MSNA-PS vs [18F]ON6
Blood	2.83 ± 0.40	13.95 ± 4.01	0.84 ± 0.12	1.83 ± 0.55	*** (0.0010)	** (0.0014)	*** (0.0005)
Plasma	4.73 ± 0.79	33.64 ± 11.55	1.51 ± 0.19	2.29 ± 0.83	** (0.0016)	** (0.0031)	** (0.0010)
Urine	482.64 ± 229.3	10.54 ± 1.74	85.05 ± 67.9	633.98 ± 283.06	* (0.0260)	* (0.0367)	* (0.0434)
Tumor	0.91 ± 0.13	1.93 ± 0.53	1.71 ± 0.54	0.69 ± 0.26	** (0.0044)	* (0.0131)	ns (0.5035)
Skin	0.78 ± 0.16	1.16 ± 0.42	2.13 ± 0.52	0.70 ± 0.22	ns (0.0842)	*** (0.0008)	** (0.0055)
Lungs	1.28 ± 0.18	11.62 ± 8.24	1.04 ± 0.11	1.32 ± 0.38	* (0.0276)	ns (0.0710)	* (0.0255)
Heart	0.78 ± 0.16	4.02 ± 1.84	1.47 ± 0.14	1.30 ± 0.22	** (0.0074)	*** (0.0004)	* (0.0191)
Thyroid	1.17 ± 0.36	2.13 ± 0.79	3.51 ± 2.15	1.68 ± 1.00	* (0.0409)	* (0.0442)	ns (0.1882)
Pancreas	0.31 ± 0.06	0.65 ± 0.15	3.99 ± 0.76	1.93 ± 0.45	** (0.0015)	*** (0.0001)	*** (0.0001)
Spleen	4.07 ± 0.61	38.19 ± 3.79	8.82 ± 1.65	0.85 ± 0.16	*** (0.0000)	*** (0.0004)	*** (0.0000)
Kidneys	3.56 ± 0.85	4.80 ± 1.18	90.99 ± 25.15	3.79 ± 1.12	ns (0.0920)	*** (0.0004)	*** (0.0004)
Adrenal gland	2.59 ± 0.15	14.47 ± 3.30	2.89 ± 1.46	0.73 ± 0.10	*** (0.0003)	ns (0.6397)	*** (0.0001)
Liver	9.22 ± 0.69	51.88 ± 9.62	41.23 ± 4.23	17.11 ± 5.6	*** (0.0001)	*** (0.0000)	* (0.0427)
Small intestine (empty)	2.39 ± 1.60	8.34 ± 4.06	9.14 ± 2.16	4.51 ± 2.16	* (0.0145)	*** (0.0005)	ns (0.6844)
Large intestine (empty)	0.52 ± 0.11	1.27 ± 0.74	4.16 ± 0.60	1.17 ± 0.28	ns (0.0547)	*** (0.0000)	*** (0.0000)
Stomach (full)	0.59 ± 0.08	3.14 ± 1.29	3.44 ± 2.16	0.63 ± 0.27	** (0.0045)	* (0.0230)	ns (0.7775)
Cecum (full)	0.27 ± 0.13	0.41 ± 0.15	1.18 ± 0.11	1.30 ± 0.31	ns (0.1533)	*** (0.0000)	*** (0.0000)
Feces	0.24 ± 0.19	0.15 ± 0.27	0.27 ± 0.39	1.27 ± 0.89	ns (0.5549)	ns (0.8721)	ns (0.5441)
White adipose tissue	0.60 ± 0.42	0.59 ± 0.39	0.53 ± 0.22	0.32 ± 0.14	ns (0.9728)	ns (0.7777)	ns (0.7511)
Ovaries	2.15 ± 0.35	6.39 ± 3.26	3.62 ± 0.93	0.85 ± 0.20	* (0.0240)	* (0.0103)	ns (0.0936)
Uterus	1.98 ± 0.84	4.28 ± 4.62	3.96 ± 1.85	0.95 ± 0.09	ns (0.2834)	ns (0.0543)	ns (0.8791)
Muscle	0.19 ± 0.07	0.19 ± 0.03	0.37 ± 0.11	0.24 ± 0.02	ns (0.9757)	* (0.0133)	* (0.0101)
Salivary glands	0.33 ± 0.04	1.04 ± 0.55	3.93 ± 0.34	0.48 ± 0.12	* (0.0246)	*** (0.0000)	*** (0.0000)
Lymph nodes	0.72 ± 0.14	3.21 ± 2.06	3.63 ± 1.21	0.76 ± 0.19	* (0.0316)	** (0.0019)	ns (0.6816)
Bone (skull)	0.63 ± 0.14	2.73 ± 1.13	1.76 ± 0.19	0.35 ± 0.08	** (0.0059)	*** (0.0000)	ns (0.0925)
Bone + marrow (femur)	1.19 ± 0.05	5.69 ± 2.35	3.50 ± 0.62	0.44 ± 0.07	** (0.0054)	*** (0.0002)	ns (0.0721)
Brain	0.06 ± 0.01	0.26 ± 0.04	0.06 ± 0.02	0.11 ± 0.04	*** (0.0000)	ns (0.9319)	*** (0.0000)
Urinary bladder	7.06 ± 7.23	10.76 ± 8.25	5.01 ± 1.44	1.91 ± 0.95	ns (0.4786)	ns (0.6123)	ns (0.1502)

ns = not significant, *p<0.05, **p<0.01, and ***p<0.001.

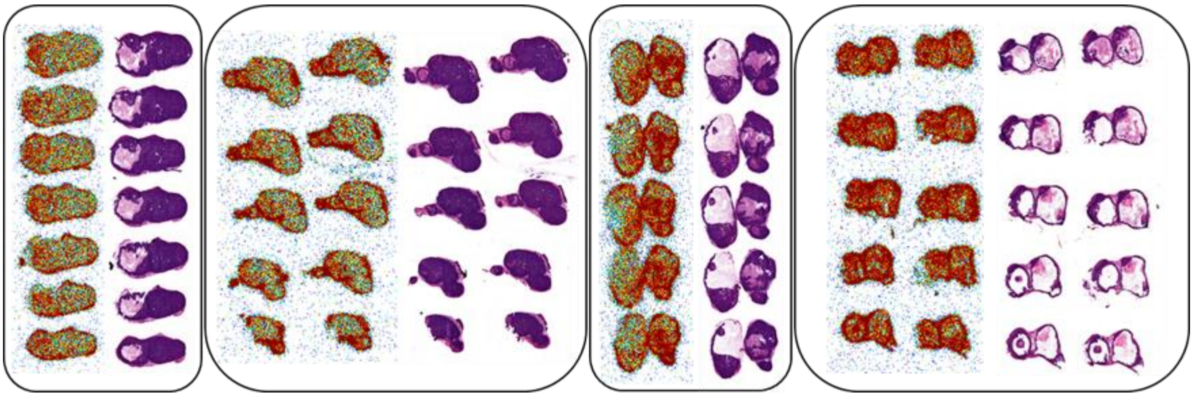

[^{18}F]MSNA-PO

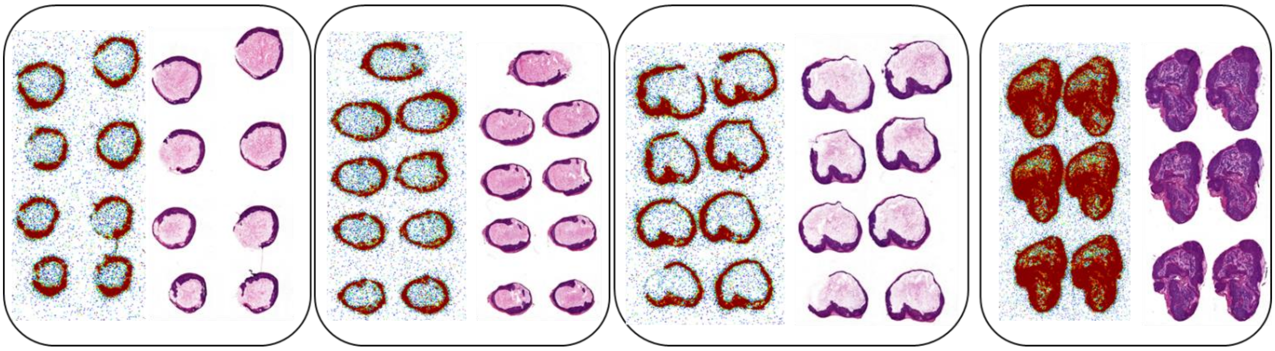

[^{18}F]MSNA-PS

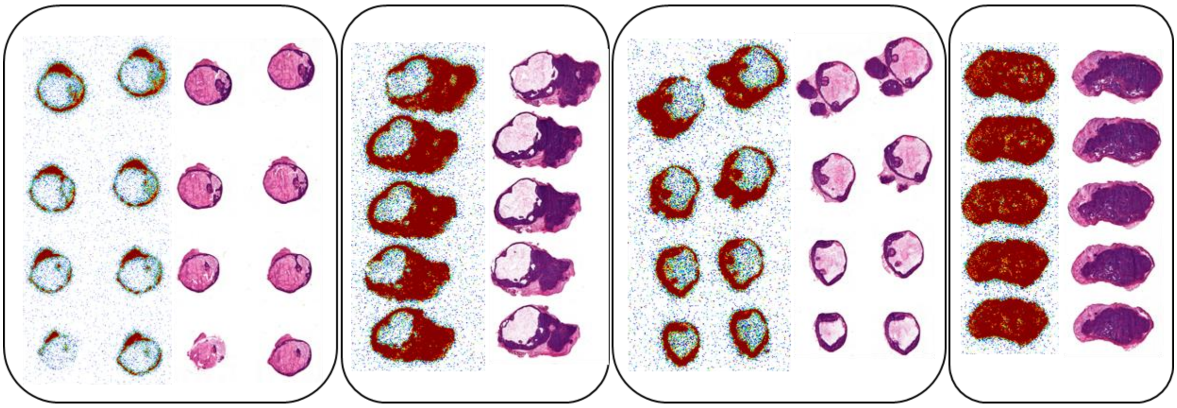

[^{18}F]ON6

Figure S11. Autoradiography (left panels) and hematoxylin-eosin staining (right panels) of 20 µm tumor cryosections 60 min after intravenous administration of **[^{18}F]MSNA-PO**, **[^{18}F]MSNA-PS** and **[^{18}F]ON6 in** HCC1954 tumor-bearing mice.

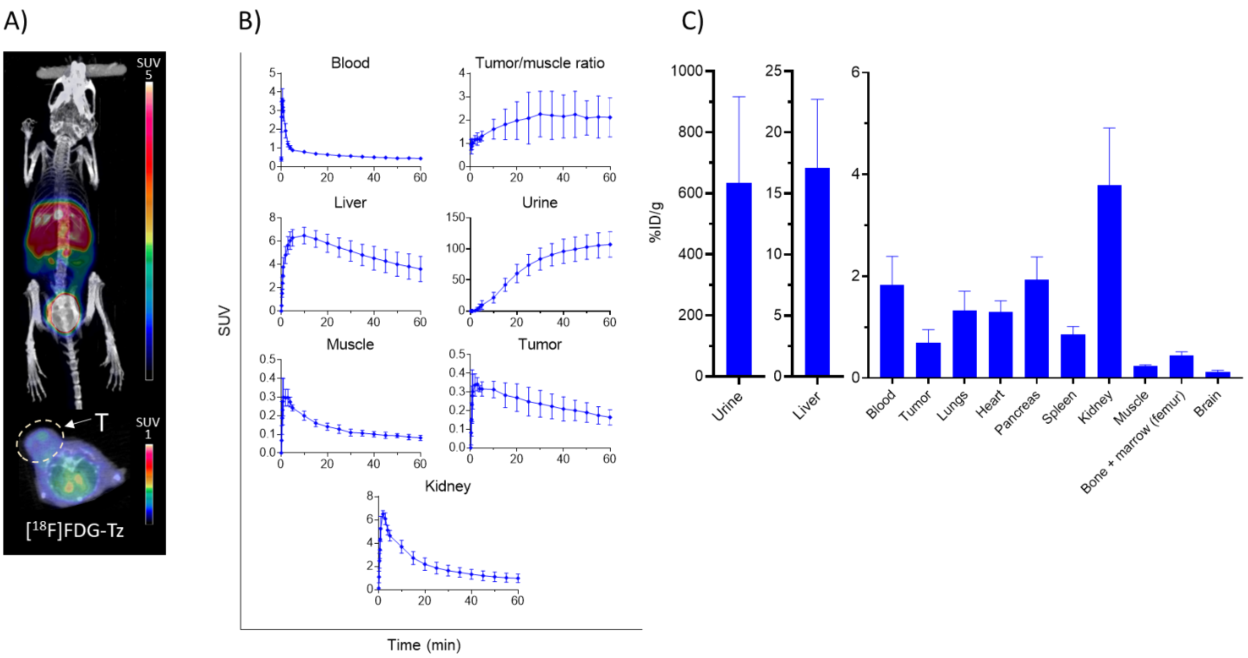

Figure S12. A) The maximum intensity projection coronal PET/CT images at 15–60 min post-injection of **[18F]FDG-Tz** in HCC1954 tumor-bearing female mice. T denotes to tumor. B) *In vivo* distribution kinetics of **[18F]FDG-Tz** in HCC1954 tumor-bearing female mice shown as time-activity curves for blood, liver, urine, muscle, tumor, and kidney expressed as standardized uptake value (SUV). C) *Ex vivo* biodistribution of **[18F]FDG-Tz** in HCC1954 tumor-bearing female mice at 60 min after injection.
